# Urinary microbiome dysbiosis is associated with an inflammatory environment and perturbed fatty acids metabolism in the pathogenesis of bladder cancer

**DOI:** 10.1186/s12967-024-05446-7

**Published:** 2024-07-05

**Authors:** Cen Wu, Xiaoyu Wei, Zhiyang Huang, Zhixiong Zheng, Wei Zhang, Jiajun Chen, Hongchang Hong, Weili Li

**Affiliations:** 1 Department of Urology, Fujian Medical University Affiliated Quanzhou First Hospital, Fujian, 362011 China; 2 Zhangjiang Center for Translational Medicine, Shanghai Biotecan Biotechnology Co., Ltd., 180 Zhangheng Road, Pudong District, Shanghai, 201204 China

**Keywords:** Bladder urothelial carcinoma, Urinary microbiome, Metabolome, Interluekin-6, Inflammation

## Abstract

**Background:**

Bladder cancer is a common malignancy with high recurrence rate. Early diagnosis and recurrence surveillance are pivotal to patients’ outcomes, which require novel minimal-invasive diagnostic tools. The urinary microbiome is associated with bladder cancer and can be used as biomarkers, but the underlying mechanism is to be fully illustrated and diagnostic performance to be improved.

**Methods:**

A total of 23 treatment-naïve bladder cancer patients and 9 non-cancerous subjects were enrolled into the Before group and Control group. After surgery, 10 patients from the Before group were further assigned into After group. Void mid-stream urine samples were collected and sent for 16S rDNA sequencing, targeted metabolomic profiling, and flow cytometry. Next, correlations were analyzed between microbiota, metabolites, and cytokines. Finally, receiver operating characteristic (ROC) curves of the urinary biomarkers were plotted and compared.

**Results:**

Comparing to the Control group, levels of IL-6 (*p* < 0.01), IL-8 (*p* < 0.05), and IL-10 (*p* < 0.05) were remarkably elevated in the Before group. The α diversity of urine microbiome was also significantly higher, with the feature microbiota positively correlated to the level of IL-6 (r = 0.58, *p* < 0.01). Significant differences in metabolic composition were also observed between the Before and Control groups, with fatty acids and fatty acylcarnitines enriched in the Before group. After tumor resection, cytokine levels and the overall microbiome structure in the After group remained similar to that of the Before group, but fatty acylcarnitines were significantly reduced (*p* < 0.05). Pathway enrichment analysis revealed beta-oxidation of fatty acids was significantly involved (*p* < 0.001). ROC curves showed that the biomarker panel of *Actinomycetaceae* + arachidonic acid + IL-6 had superior diagnostic performance, with sensitivity of 0.94 and specificity of 1.00.

**Conclusions:**

Microbiome dysbiosis, proinflammatory environment and altered fatty acids metabolism are involved in the pathogenesis of bladder cancer, which may throw light on novel noninvasive diagnostic tool development.

**Supplementary Information:**

The online version contains supplementary material available at 10.1186/s12967-024-05446-7.

## Introduction

Bladder cancer is the tenth leading cancer type globally with more than 573,000 new cases in 2020 [1]. In U.S., the estimated incidence is over 80,000 of both sexes in 2024, with approximately three quarters occurring in men while one quarter occurring in women [2]. In China, bladder cancer is also common which ranked the eighth in morbidity and mortality among all cancer types in males [3]. Painless haematuria, microscopic or visible, is an alerting sign for bladder cancer. Medical attention should be paid to those with haematuria, but actually the urological referral rate of these patients remains at a low level [4]. What’s more, the recurrence rate of bladder cancer at 5 years can be as high as 70% [5], making it a heavy disease burden to the society. Cystoscopy is the gold standard for both diagnosis and recurrence surveillance, but it is invasive and usually costly due to repeated usage. Urine is in continuous contact with bladder cancer tissue and contains information both on tumor environment and tumor itself, making it a promising tool to be developed for disease screening, diagnosis, and recurrence monitoring. Urine cytology is a frequently used marker that has been approved to apply in adjuvant with cystoscopy, but its prominent defect in sensitivity has restricted its role in detecting low-grade diseases. In addition, many urine assays based on proteins, DNA, RNA, and other biomarkers, have also been developed [6]. However, they encounter many other limitations, such as low specificity and false positive results [7, 8]. Therefore, a more robust urinary biomarker or panels designed for early diagnosis and recurrent surveillance will be of great clinical value to improve bladder cancer management and patient’s outcomes.

That urinary tract was sterile had been a long-held doctrine until several studies found a unique urinary microbiota harbors in it [9–11]. Differences in urinary microbiome between bladder cancer subjects and the healthy controls have been extensively studied [12, 13]. For example, *Actinomyces*, *Achromobacter*, *Brevibacterium*, and *Brucella* were reported to be more abundant in bladder cancer urines when compared to healthy controls [13]. As for microbiome diversity, several studies [12, 14, 15] reported a significantly higher α diversity in bladder cancer patients, which indicated an increased species diversity in cancer group. Unlike gut microbiome studies, the microbial diversity was inconsistent among urinary tract disorders and Zeng et al. [15] even correlated low alpha diversity (especially Shannon index) to a prolonged recurrence-free survival. The inconsistence across studies is partly due to the variance in sample types used in their research. The most studied sample types included void mid-stream urine, cathedral urine, and tumor tissues, each with its own advantages and disadvantages. But void mid-stream urine is the most accessible type and can be exploited as a minimal invasive tool for cancer screening, diagnosing as well as recurrence surveillance. Nevertheless, though the urinary microbiome is an increasingly understood area, the findings on its role in bladder cancer pathogenesis remain insufficiently elucidated. Some studies proposed that chronic inflammation, caused by urinary microbial dysbiosis, might lead to cancer initiation [16, 17]. During this process, the translocation of bacterial could activate Toll-like receptors (TLRs), followed by the activation of inflammation-associated pathways, including NF-kB and Janus-activated kinase (JAK)-STAT3 signaling pathways, which lead to upregulation of proinflammatory cytokines and finally induce an inflammatory bladder environment [16]. Particularly, the proinflammatory cytokine IL-6 was reported to be associated with initiation and progression of bladder cancer [18, 19]. But it remains unclear what taxa is responsible for chronic inflammation. On the other hand, the immunotherapy with intravesical Bacillus Calmette–Guérin (BCG) vaccine yields generally good outcomes for many non-muscle invasive bladder cancer (NMIBC) patients, which signifies that the immune-inflammation system, activated by the microbe in the vaccine, could play a positive effect against bladder tumors [20]. The contrasting findings reminded us that a more overall picture should be painted on the urinary microbiome and its functions.

As living organisms, urinary microbes exchange materials by “eating”, producing, and releasing substances into the urine. Thus, the urinary metabolome is a reservoir that contains metabolites produced by normal urothelial cells, tumor cells, and microbes, including amino acids, organic acids, carbohydrates, and so on. Accumulating research has explored urinary metabolomic biomarkers to differentiate bladder cancer patients from non-cancerous populations. For example, Nizioł et al. [21] reported a series of metabolites detecting bladder cancer with sensitivity ranging from 80 to 90% and specificity from 81 to 90%. Lin et al. [22] also found eight metabolites with sensitivity varying between 67 and 92% and specificity between 75 and 100%. Similar studies abound, but there is a dearth of research that exploits void urine to unveil the underlying mechanism of bladder cancer from a multi-dimensional perspective. Integrative studies that combined urinary microbiome and metabolome could provide extensive additional information about the tumorigenic liquid environment that tumor tissue reside in, and useful urinary biomarkers or marker panels might be discovered in time.

Urinary microbiome and urinary metabolome have been studied extensively, and some microbiota and metabolites have been proposed as biomarkers for bladder cancer [23–26]. However, the diagnostic performance of these single biomarkers was limited, and the potential mechanism and function of urinary microbiome remain insufficiently elucidated. In this study, we conducted an integrated exploration based on microbiomics, targeted metabolomics, and cytokine profiling to reveal the pathogenesis of bladder cancer. First, 16S rDNA sequencing, ultra-performance liquid chromatography coupled to tandem mass spectrometry (UPLC-MS/MS), and flow cytometry were performed with void mid-stream urine samples collected from non-cancerous and bladder cancer patients pre- and post-operation, respectively. Next, Spearman correlation was analyzed between microbiota and cytokines, as well as between microbiota and metabolites. Finally, receiver operating characteristic (ROC) curves were plotted and compared on the biomarkers generated in the steps above.

## Materials and methods

### Study design and sample collection

From May 2021 to November 2022, eligible participants were screened and recruited into the Before group at the Department of Urology of Quanzhou First Hospital (Fujian Province, China). Inclusion criteria were as follows: (1) 40–80 years old; (2) diagnosed with primary or recurrent bladder urothelial cancer, single or multiple tumors; (3) no therapy was used at the time of enrollment. Exclusion criteria were as follows: (1) lack of informed consent; (2) absence of histological confirmation; (3) presence of other malignancies. Meanwhile, age- and sex-matched subjects who were ruled out for the presence of urological malignancies or other alterations by ultrasound scan were recruited into the Control group. Routine blood tests and urinalysis, including urine specific gravity, were measured by laboratory standardized methods, and midstream urine samples were collected from all participants in sterile 50 mL centrifuge tubes and immediately frozen at − 80 °C until analyzed. Besides, ten patients from the Before group who underwent transurethral resection of bladder tumors (TURBT) were further selected into the After group, midstream urine samples of these patients were again collected 2–3 weeks after the surgery and stored at − 80 °C. When completing sample collection of three groups, all samples were sent for analysis and tested in the same batch. Written informed consent was obtained from all participants prior to sample collection. The study was performed according to the Declaration of Helsinki and was approved by the Ethics Committee of Quanzhou First Hospital (#2020-226).

### Urine microbiome analysis with 16S rDNA sequencing

First, urine samples were thawed on an ice bath, and 1 mL of each sample was taken for DNA extraction. Total bacterial genomic DNA was extracted by the PowerMax DNA isolation kit (MoBio Laboratories, Carlsbad, CA, USA) according to the manufacturer’s instructions and stored at − 20 °C for further analysis. The V4 region of the bacterial 16S rRNA genes was amplified using the forward primer 515F (5′- GTGCCAGCMGCCGCGGTAA-3′) and the reverse primer 806R (5′-GGACTACHVGGGTWTCTAAT-3′). Paired-end barcodes of 6-bp for each sample were integrated into the TrueSeq adaptors for multiplex sequencing. The PCR reaction mix consisted of 25 μL of Phusion High-Fidelity PCR Master Mix, 3 μL (10 uM) of each Forward and Reverse primer, 10 μL of DNA Template, 3 μL of DMSO, and 6 μL of ddH2O. Thermal cycling procedures were set as follows: initial denaturation at 98 °C for 30 s, followed by 25 cycles of denaturation at 98 °C for 15 s, annealing at 58 °C for 15 s, and extension at 72 °C for 15 s, with a final extension of 1 min at 72 °C. Then, the Agencourt AMPure XP Beads (Beckman Coulter, Indianapolis, IN) and the PicoGreen dsDNA Assay Kit (Invitrogen, Carlsbad, CA, USA) were used to purify and quantify the PCR amplicons. Finally, amplicons were pooled in equal amounts and sequenced via the pair-end 2 × 150 bp Illumina NovaSeq6000 platform.

During the bioinformatic analysis procedure, raw sequencing reads that exactly matched the barcodes were allocated to the corresponding samples and identified as valid sequences. The following low-quality sequences were filtered out: sequences that (1) had a length of < 150 bp; (2) had average Phred scores of < 20; (3) contained ambiguous bases; (4) contained mononucleotide repeats of > 8 bp. Vsearch V2.4.4 was used to assemble paired-end reads, and Vsearch v2.15.0 was applied to pick the Operational Taxonomic Units(OTUs). The Quantitative Insights Into Microbial Ecology (QIIME2, v2020.6) pipeline and R packages (v3.2.0) were utilized to process the OTUs and sequencing data. OTU taxonomic classification was performed by searching against the SILVA138 database. OTU-level alpha diversity indices, including Chao1 richness, Shannon diversity index, and Simpson index, and beta diversity, including UniFrac distance metrics, were performed with QIIME2 and visualized to determine the structural variation of microbial communities across samples. Ranked abundance curves were generated to compare the richness and evenness of OTUs for all samples. Linear discriminant analysis effect size (LEfSe) was performed to explore differentially abundant taxa across groups with the default parameters. Pairwise comparisons of differences in the Unifrac distances were determined by performing Student’s t-test and the Monte Carlo permutation test with 1000 permutations. The variance of microbiota structure among groups was assessed by using Permutational multivariate analysis of variance (PERMANOVA) with R package “vegan”. Differences in taxa abundances at the phylum, class, order, family, and genus levels were calculated by the Kruskal test.

### Urine targeted metabolites profiling with UPLC-MS/MS

Targeted metabolomics was performed using the Q300 Metabolite Array Kit (Metabo-Profile, Shanghai, China). All standard metabolites were purchased from Sigma-Aldrich (St. Louis, MO, USA), Steraloids Inc. (Newport, RI, USA), Thermo-Fisher Scientific (FairLawn, NJ, USA), and TRC Chemicals (Toronto, ON, Canada) and were accurately weighed and prepared in water, methanol, sodium hydroxide solution, or hydrochloric acid solution to obtain individual stock solutions with a concentration of 5.0 mg/mL. Ultrapure water was obtained from a Mill-Q Reference system equipped with an LC–MS Pak filter (Millipore, Billerica, MA, USA). First, urine samples were thawed on an ice-salt bath to minimize sample degradation. Then, 25 μL of urine was added to a 96-well plate and transferred to the Eppendorf epMotion Workstation (Eppendorf Inc., Humburg, Germany). After adding 120 μL ice-cold methanol with partial internal standards to each sample, the plate was centrifuged at 4000*g* for 30 min. Next, 30 μL of supernatant was transferred to a clean 96-well plate, and 20 μL of freshly prepared derivative reagents was added to each well, followed by derivatization at 30 °C for 60 min. Finally, the plate was sealed for LC–MS analysis after derivatization and dilution. For quality control (QC), QC samples were prepared by mixing the test samples and injected at regular intervals throughout the analytical run. In addition, reagent blank samples were processed during the same procedures and were also used to wash the column and reduce cumulative matrix effects. Besides, the samples were analyzed in group pairs randomly to diminish analytical bias. The QC samples, blank samples, and calibrators, consisting of a blank sample, a zero sample, and seven concentrations of metabolites, were analyzed within the entire analytical process.

Targeted metabolites in urine samples were quantitated with UPLC-MS/MS system (ACQUITY UPLC-Xevo TQ-S, Waters Corp., Milford, MA, USA). The instrument settings can be found in Supplementary Table 1. The TMBQ software (v1.0, Metabo-Profile, Shanghai, China) was utilized to process the raw data files generated by UPLC-MS/MS by performing peak integration, calibration, and quantitation for each metabolite. Before analysis, the metabolomic profile of urine samples was normalized to specific gravity as previously described [27]. Then, the in-house platform iMAP (v1.0, Metabo-Profile, Shanghai, China) was applied for statistical analyses. A calibration curve was plotted to yield a linear model described as y = ax + b, where the analyte concentration (x) of unknown samples can be calculated from this equation. Next, multivariate statistical analyses and univariate statistical analyses were performed via statistical analysis software packages in R studio (http://cran.r-project.org/), including principal component analysis (PCA), partial least square discriminant analysis (PLS-DA), orthogonal partial least square discriminant analysis (OPLS-DA), student t-test, Mann Whitney (U-test), ANOVA, and Spearman rank correlation analysis. The Sankey plot of relationships between microbiota and metabolites were performed via MetOrigin [28] (https://metorigin.met-bioinformatics.cn/).

### Urine cytokines detection by flow cytometry

First, urine samples were thawed on an ice bath followed by centrifugation at 400*g* for 5 min at 4 °C to remove cellular debris. Then, 50 µL of supernatant of each sample was taken to incubate with capture-bead suspensions and fluorescent-labeled antibodies with EasyMagPlex Human Cytokine 12 Plex Kit (category number: 281601HN, Wellgrow, Shenzhen, China). Meanwhile, a standard curve was also prepared according to manufacturer’s instructions. Next, flow cytometry was performed on DxFLEX (Beckman Coulter, Germany) with a termination condition of capturing 120 events at the P4 gate or recording for 600 s. Finally, WellEasy CKAS1.0.2 was applied to process the data and calculate the concentrations of IL-1β, IL-2, IL-4, IL-5, IL-6, IL-8, IL-10, IL-12p70, IL-17, TNF-α, IFN-γ, IFN-α.

### Statistical analysis

Normally distributed continuous variables were displayed as mean ± standard deviation, and comparisons between two groups were calculated with Student’s t-test or paired t-test; non-normally distributed continuous variables were displayed as median (Q1, Q3), and comparisons between two groups were calculated by Mann–Whitney U test; categorical variables were displayed as number (%), and comparisons between two groups were calculated by chi-square test. One-way ANOVA or Kruskal–Wallis test was used to compare differences among three groups. The Delong test [29] was performed for comparisons between ROC curves.

## Results

**Fig. 1:**
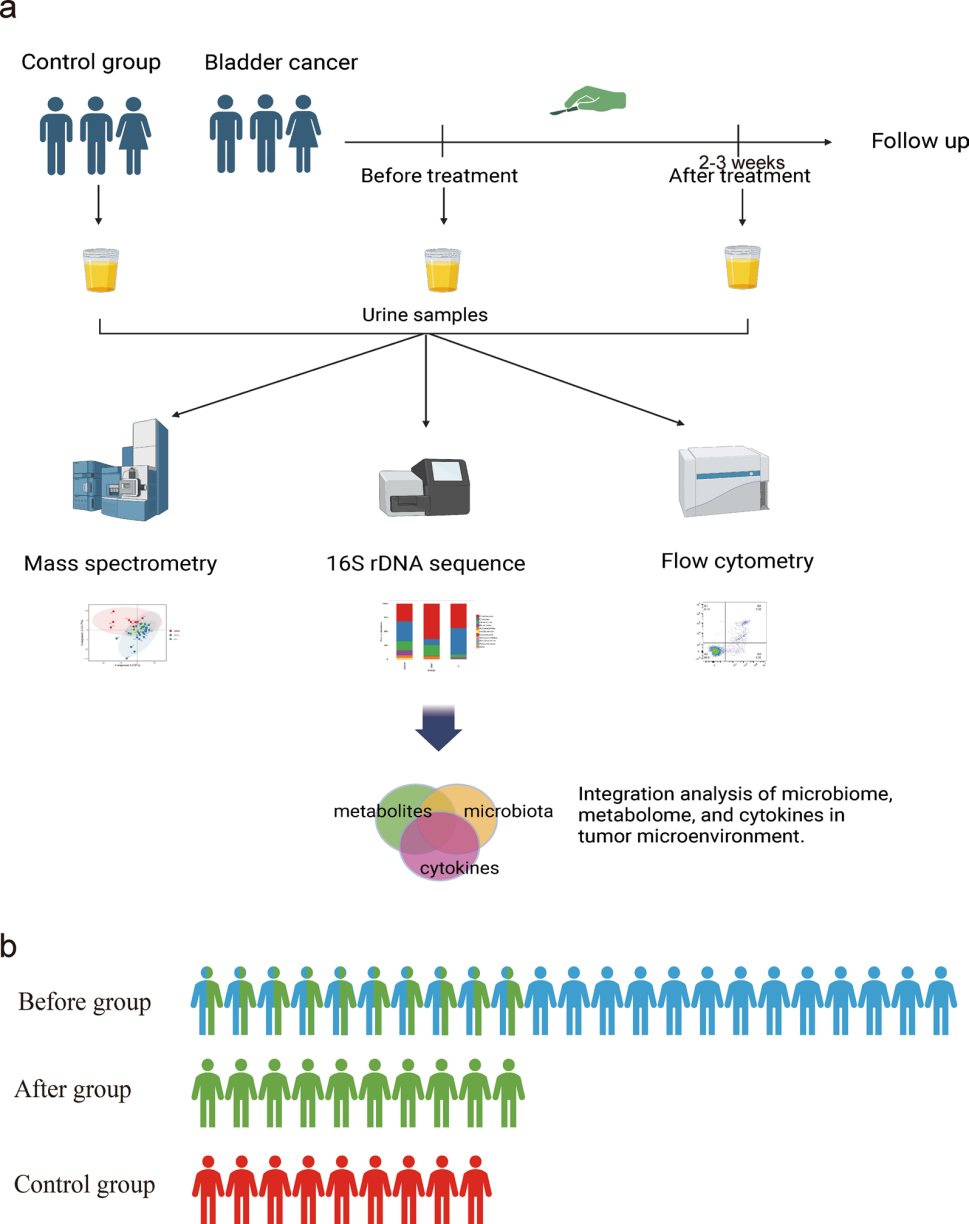
**a** Flowchart of the study design; **b** participants distribution of three groups

A total of twenty-three eligible bladder urothelial carcinoma patients and nine non-cancerous participants were enrolled in this study. The clinical characteristics are displayed in Table 1. The age, sex, BMI, and history of urolithiasis were similar between the two groups. The proportions of smokers and individuals with a history of urinary tract infection (UTI) were significantly higher in the Before group than in the Control group (*p* < 0.05; *p* < 0.01). Most subjects (78%) in the Before group underwent TURBT. Among these, ten subjects were enrolled in the After group, whose urine samples were collected within three weeks after TURBT surgery. The flowchart of the study design was displayed in Fig. 1.

**Table 1:** Clinicopathological characteristics of all subjects included in this study

	Before group (n = 23)	Control group (n = 9)	*p-*value
Age, y	64.30 ± 11.18	56.44 ± 12.89	0.12
Sex, n (%)			1.00
Male	20 (86.96%)	8 (88.89%)	
Female	3 (13.04%)	1 (11.11%)	
BMI, kg/m^2^	23.46 ± 3.36	23.81 ± 2.59	0.79
Smoking history, n (%)			0.02
Smoker	17 (73.91%)	2 (22.22%)	
Nonsmoker	6 (26.09%)	7 (77.78%)	
UTI history, n (%)			0.006
Yes	18 (78.26%)	2 (22.22%)	
No	5 (21.74%)	7 (77.78%)	
Urolithiasis, n (%)			1.00
Yes	3 (13.04%)	1 (11.11%)	
No	20 (86.96%)	8 (88.89%)	
Comorbidities, frequencies			–
Hypertension	15	3	
Diabetes mellitus	3	1	
Cardiovascular diseases	2	0	
Hyperplasia prostate	4	3	
Others	1	1	
TNM stage, n (%)		–	–
0–I	13 (56.52%)		
II	3 (13.04%)		
III	3 (13.04%)		
IV	0		
Unknown/Unclassified	4 (17.40%)		
WHO grade, n (%)		–	–
1	6 (26.09%)		
2	6 (26.09%)		
3	7 (30.43%)		
Unknown/Unclassified	4 (17.39%)		
Treatment		–	–
TURBT	18 (78.26%)		
Laparoscopic radical cystectomy	2 (8.70%)		
Laparoscopic partial cystectomy	3 (13.04)		

BMI: Body mass index; UTI: Urinary tract infection; TNM: Tumor Node Metastasis classification; WHO: World Health Organization; TURBT: Transurethral resection of bladder tumors. *p* was calculated by Student’s t-test or Fisher’s exact test

### Urine cytokine analysis revealed an inflammatory environment in bladder cancer patients which cannot be reversed by tumor resection

The levels of proinflammatory cytokines, including IL-6 and IL-8, were significantly increased in the Before group compared to the Control group (*p* < 0.01; *p* < 0.05), while the anti-inflammatory cytokine, IL-10, was also upregulated in the Before group (*p* < 0.05). The levels of other cytokines, such as IL-1β, IL-2, IL-4, IL-5, IL-12p70, IL-17, TNFα, IFNα, and IFNγ, were similar between the two groups (Table 2). However, after tumor resection, no remarkable changes in cytokine levels were observed in these patients, with the IL-6 level in the After group still higher than that of Control group (*p* < 0.05) (Supplementary Table 2 and 3).

**Table 2:** Differences in urine cytokine levels between the Control group and the Before group

	Before group (n = 23)	Control group (n = 9)	*p-*value
IL-1β (pg/mL)	17.86 (4.95, 34.57)	8.30 (4.74, 48.04)	0.66
IL-2 (pg/mL)	1.82 ± 1.09	1.42 ± 0.75	0.32
IL-4 (pg/mL)	9.64 ± 5.64	7.44 ± 5.67	0.34
IL-5 (pg/mL)	10.64 ± 5.34	7.97 ± 3.69	0.18
IL-6 (pg/mL)	12.75 (7.80, 34.52)	6.36 (5.39, 8.26)	0.00
IL-8 (pg/mL)	54.86 (4.53, 160.17)	6.38 (3.44, 23.89)	0.03
IL-10 (pg/mL)	6.00 (4.08, 7.97)	3.39 (2.25, 5.57)	0.02
IL-12p70 (pg/mL)	14.33 (5.11, 22.29)	6.99 (3.42, 28.32)	0.57
IL-17 (pg/mL)	24.77 (9.02, 32.19)	14.17 (10.61, 25.11)	0.49
TNF-α (pg/mL)	5.92 (2.67, 7.71)	3.23 (1.19, 6.35)	0.20
IFN-α (pg/mL)	1.50 (0.64, 2.40)	0.83 (0.59, 1.40)	0.10
IFN-γ (pg/mL)	5.72 ± 3.01	3.81 ± 2.62	0.11

IL: interleukin; TNF: tumor necrosis factor; IFN: interferon. *p* was calculated by Student’s t-test or Mann–Whitney U test

### Diversity and abundance of urine microbiota increased in the Before group and was positively correlated with urine cytokine levels

**Fig. 2:**
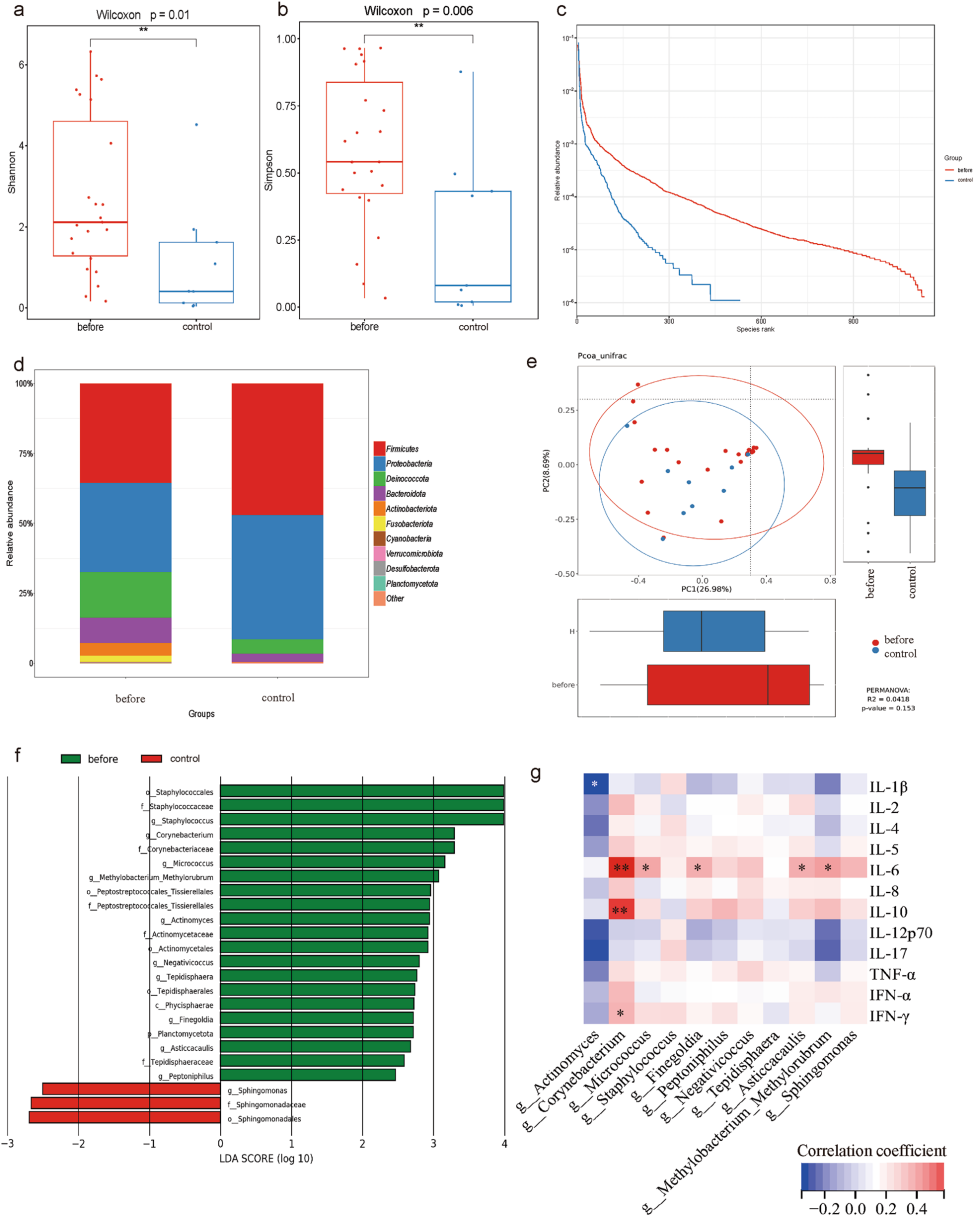
Urine microbiome characteristics and association with urine cytokines. **a** Shannon index and Simpson index (**b**) indicated a significant higher α diversity of urine microbiome in the Before group compared to the Control group (Wilcoxon test *p* < 0.05; *p* < 0.01); **c** Rank-abundance curve showed differences in richness and evenness between two groups; **d** Taxonomy bar plot displayed dominant phyla between two groups; **e** PCoA analysis showed no significant difference in β diversity between the Before group and the Control group (*p* > 0.05). **f** LEfSe analysis identified a series of feature microbiota for the Before group; **g** Spearman correlation analysis revealed a positive relationship between the feature microbiota and the level of urine cytokines. *p < 0.05; **p < 0.01. IL: interleukin; TNF: tumor necrosis factor; IFN: interferon

The α diversity indices of the urine microbiome, including the Shannon index and Simpson index, were remarkably increased in the Before group (p < 0.05; p < 0.01) when compared to the Control group (Fig. 2a, b), but the other indexes were not significantly different (Supplementary Fig. 1). Besides, the rank-abundance curve indicated a higher richness and evenness of the urine microbiome in the Before group (Fig. 2c). Microbiome composition analysis showed that phyla *Firmicutes* and *Proteobacteria* dominated in the Control group, while in the Before group, more phyla were observed, including phylum *Deinococcota*, *Bacteroidota*, *Actinobacteriota*, *Fusobacteriota*, and others (Fig. 2d). Principal Co-ordinates Analysis (PCoA) showed that the difference in β diversity between the Before group and the Control group was not significant (*p* > 0.05) (Fig. 2e). LEfSe analysis identified feature microbiota for the Before group, including *o_Staphylococcales, f_Staphylococcaceae, g_Staphylococcus, g_Corynebacterium, f_Corynebacteriaceae, g_Micrococcus, g_Methylobacterium_Methylorubrum, o_Peptostreptococcales_Tissierellales, f_Peptostreptococcales_Tissierellales, g_Actinomyces, o_Actinomycetales, f_Actinomycetaceae, g_Negativicoccus, g_Tepidisphaera, o_Tepidisphaerales, c_Phycisphaerae, g_Finegoldia, p_Planctomycetota, g_Asticcacaulis, f_Tepidisphaeraceae,* and *g_Peptoniphilus* (Fig. 2f). Finally, the Spearman correlation analysis showed that the abundance of *g_Corynebacterium* was positively correlated with the levels of IL-6 (r = 0.58, *p* < 0.01), IL-10 (r = 0.56, *p* < 0.01), and IFNγ (r = 0.35, *p* < 0.05) (Fig. 2g)*.* Other feature microbiota, such as *g_Micrococcus* (r = 0.38, *p* < 0.05), *g_Finegoldia* (r = 0.36, *p* < 0.05), *g_Asticcacaulis* (r = 0.36, *p* < 0.05), and *g_Methylobacterium_Methylorubrum* (r = 0.40, *p* < 0.05), were also positively related to the IL-6 level.

### Urine metabolome differed in the Before group with fatty acids and fatty acylcarnitines remarkably upregulated

**Fig. 3:**
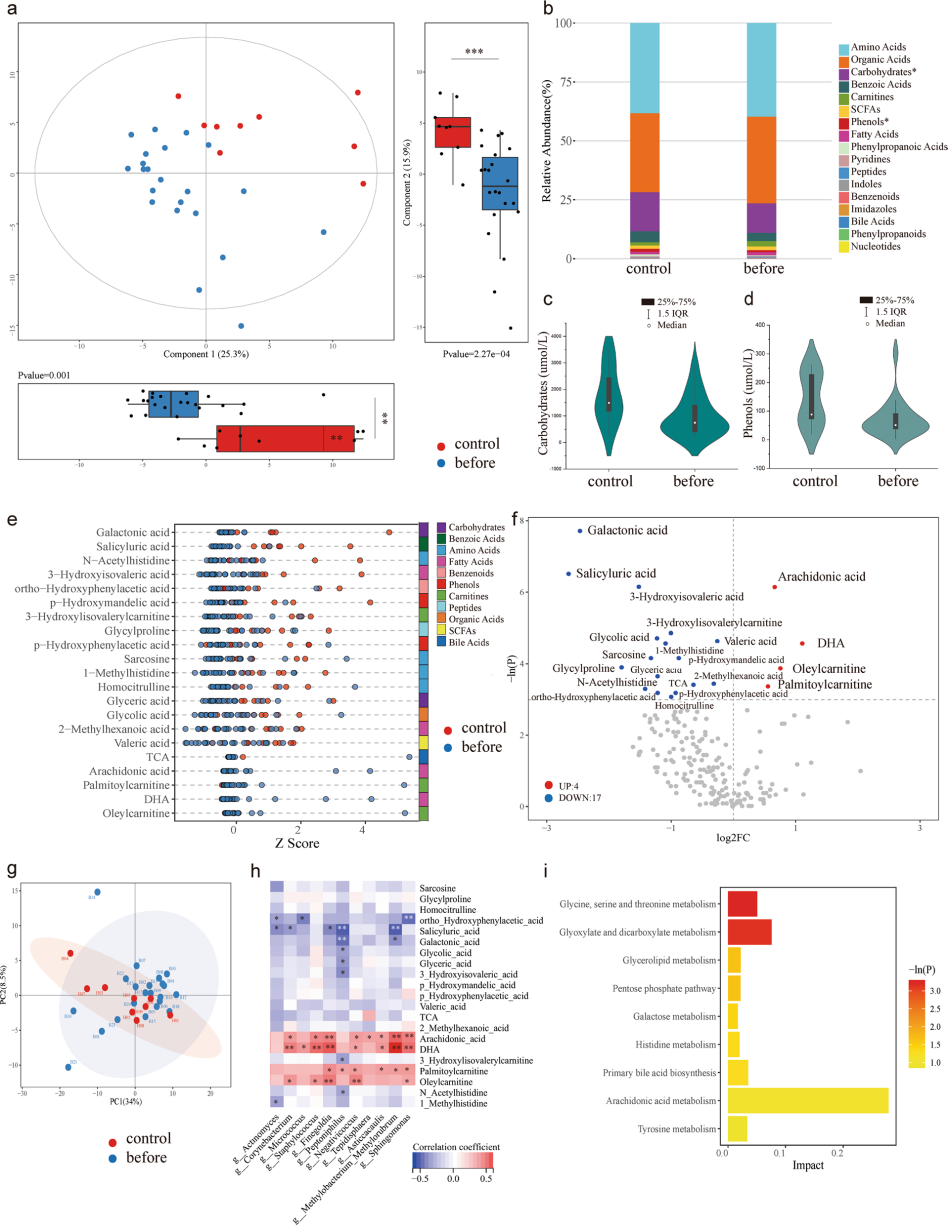
Urine metabolome characteristics and association with urine cytokines. **a** PLS-DA model showed metabolic discrimination between Before and Control groups in Component 1 (25.3%, *p* < 0.01) and Component 2 (15.9%, *p* < 0.001); **b** comparison of the relative abundance of metabolite classes between Before group and Control group, which showed that **c** carbohydrates and **d** phenols were higher in the Control group (Wilcoxon test *p* < 0.05; *p* < 0.05); **e** Z score plot and **f** volcanic plot of differentially expressed metabolites, a single dot represent a patient in Z score plot or a metabolite in volcanic plot; **g** Metabolites clusters of the Before group and the Control group with PCA analysis; **h** Spearman correlation analysis revealed a close relationship between the metabolites and urine microbiota; **i** Pathway enrichment analysis of the differentially expressed metabolites. *p < 0.05; **p < 0.01. SCFAs: short chain fatty acids; TCA: taurocholic acid; DHA: docosahexaenoic acid; IL: interleukin; TNF: tumor necrosis factor; IFN: interferon

There were significant differences in metabolic composition (component1 25.3% *p* < 0.01; component2 15.9% *p* < 0.001) between the Before group and Control group according to PLS-DA model (Fig. 3a). The carbohydrates and phenols were significantly higher in the Control group (*p* < 0.05; *p* < 0.05) (Fig. 3b–d). Univariate analysis identified 17 downregulated and 4 upregulated metabolites in the Before group, including arachidonic acid, DHA, palmitoylcainitine, and oleylcarnitine (Fig. 3e, f). The metabolites clusters of the two groups with Principal Component Analysis (PCA) were shown in Fig. 3g, but no significant difference was observed (PC1 34%, *p* > 0.05; PC2 8.5%, *p* > 0.05). Spearman correlation analysis revealed generally positive correlations between the feature microbiota and the 4 upregulated metabolites in Before group (Fig. 3h). Predicted pathway of the differentially expressed metabolites showed that Glyoxylate and dicarboxylate metabolism might be involved in the pathogenesis of bladder cancer (Fig. 3i).

### After tumor removal, fatty acylcarnitines significantly decreased but urine microbiota remained similar

**Fig. 4:**
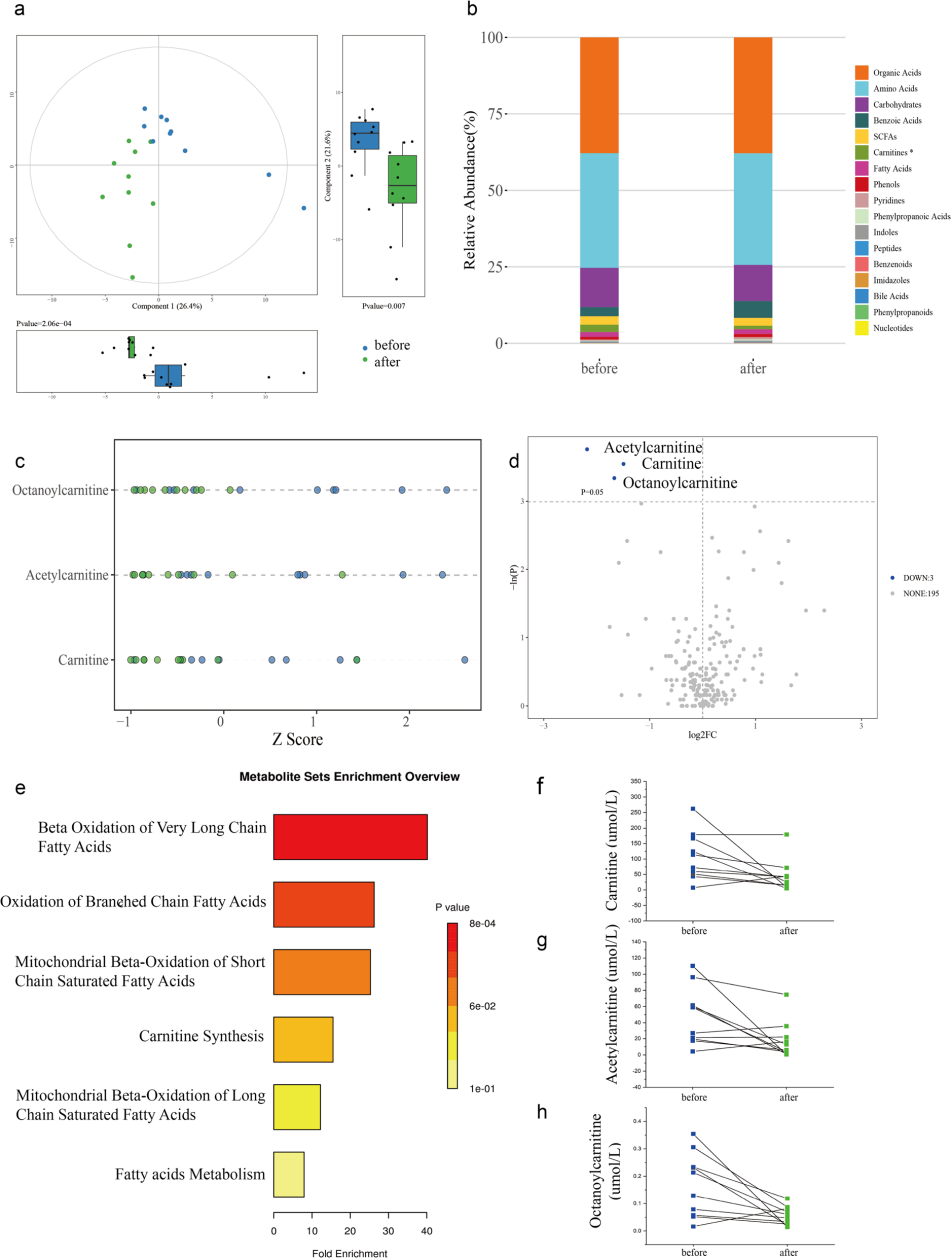
Urine metabolome changes after tumor resection. **a** PLS-DA model showed metabolic discrimination after tumor resection in Component 1 (26.4%, *p* < 0.001) and Component 2 (21.6%, *p* < 0.01); **b** comparison of the relative abundance of metabolite classes between Before group and After group; **c** Z score plot and **d** volcanic plot of differentially expressed metabolites, a single dot represent a patient in Z score plot or a metabolite in volcanic plot; **e** pathway enrichment of differentially expressed metabolites; **f** carnitine, **g** acetylcarnitine, and **h** octanoylcarnitine levels remarkably dropped after tumor resection (paired t test *p* < 0.05; *p* < 0.05; *p* < 0.05). SCFAs: short chain fatty acids

As shown in Fig. 4a, the PLS-DA model indicated a remarkable variance in urine metabolites between the Before group and After group (component1 26.4% *p* < 0.001; component2 21.6% *p* < 0.01). According to univariate analysis, the proportions of carnitines, in particular, octanoylcarnitine, acetylcarnitine, and carnitine, were greatly reduced after tumor removal (*p* < 0.05;* p* < 0.05;* p* < 0.05) (Fig. 4b–d, f–h). Metabolism pathway enrichment analysis of the differentially expressed metabolites showed that beta-oxidation of very long-chain fatty acids and oxidation of branched-chain fatty acids were associated with bladder cancers (Fig. 4e). However, there was no significant difference in the diversity and composition of microbiota between the Before group and After group (Supplementary Fig. 2).

**Fig. 5:**
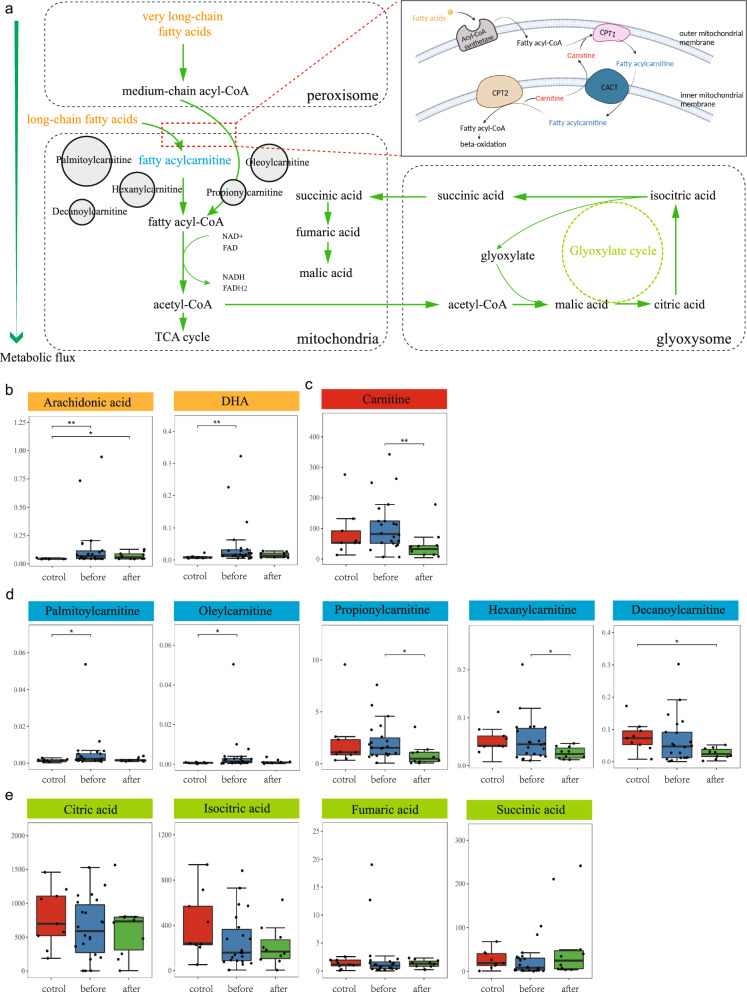
Potential pathways involved in the pathogenesis of bladder urothelial carcinoma. **a** Schematic diagram of the fatty acids transportation, beta-oxidation, and glyoxylate cycle. Prior to being shuttled into the mitochondria, fatty acids will be activated into fatty acyl-CoA by fatty acyl-CoA synthetase, followed by converted to fatty acylcarnitines by the CPT1 located on the outer mitochondrial membrane. Then, CACT on the inner membrane shuttles fatty acylcarnitines into the mitochondrial matrix, where the CPT2 on the matrix side of the inner membrane reconverts acylcarnitine into acyl-CoA for further oxidation; **b** fatty acids, **c** carnitines, **d** fatty acylcarnitines, and **e** metabolites in glyoxylate cycle changes among patients before and after tumor resection when compared to the Control group (Wilcoxon test *p < 0.05; **p < 0.01). CPT1: carnitine palmitoyltransferase I; CPT2: carnitine palmitoyltransferase II; CACT: carnitine-acylcarnitine translocase; DHA: docosahexaenoic acid; TCA: tricarboxylic acid cycle. Figure generated by Biorender (https://biorender.com/)

The heatmaps of the metabolites of all samples were displayed in Supplementary Fig. 3a, b. The Sankey maps of the production and inhibition relationships between featured microbiota and metabolites were shown in Supplementary Fig. 3c–f. The potential metabolic pathways of host cells and urine microbiota are integrated in Fig. 5a, which included fatty acid transportation, beta-oxidation, and glyoxylate cycle. Typically, the Before group had higher levels of fatty acids, carnitine, and fatty acylcarnitines than the others. For example, arachidonic acid, DHA, palmitoylcarnitine, and oleylcarnitine were more abundant than in the Control group (*p* < 0.01; *p* < 0.01; *p* < 0.05; *p* < 0.05), while carnitine, propionylcarnitine, and hexanylcarnitine were higher than After group (*p* < 0.01; *p* < 0.05; *p* < 0.05) (Fig. 5b–d). Although no significant difference was observed, the levels of citric acid, isocitric acid, fumaric acid and succinic acid were slightly lower in the Before group than in the others (Fig. 5e).

### Combination of urine cytokine, microbiota and metabolite demonstrated excellent diagnostic efficiency in predicting bladder cancers from noncancerous population

**Fig. 6:**
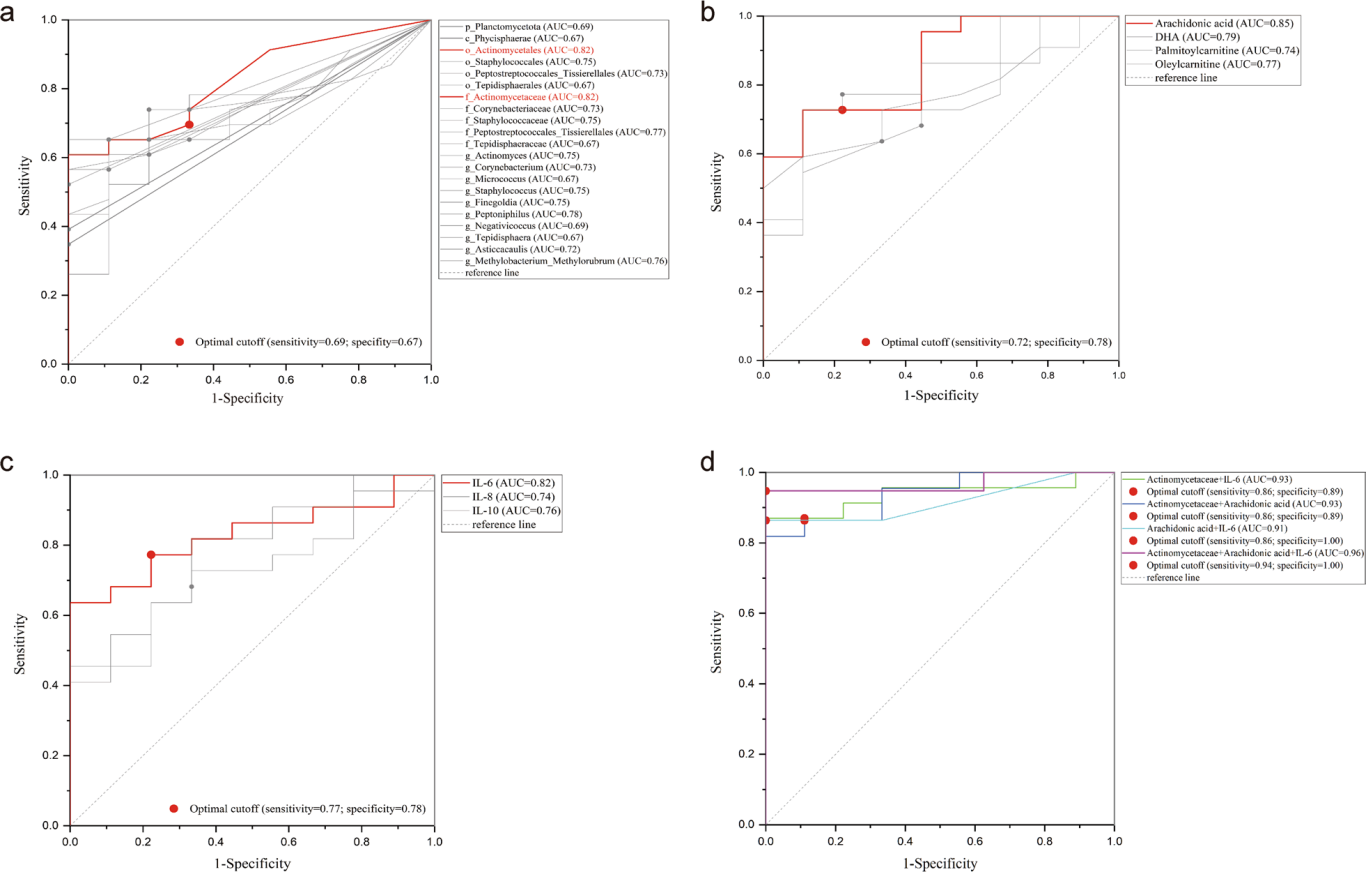
ROC curves of **a** the feature microbiota, **b** differentially expressed metabolites, **c** upregulated cytokines, and **d** the combinations of biomarkers with largest AUC value for prediction bladder urothelial cancer. ROC: receiver operating characteristic curve; AUC: area under the curve; DHA: docosahexaenoic acid; IL: interleukin; TNF: tumor necrosis factor; IFN: interferon

To find novel biomarkers for prediction bladder cancers, ROC curves were plotted for feature microbiota, metabolites and differentially expressed cytokines independently and in combination. For urine microbiota, the AUC of *o_Actinomycetales* and *f_Actinomycetaceae* reached 0.82 (95% CI 0.64–1.00), with sensitivity of 0.69 and specificity of 0.67 (Fig. 6a). Regarding urine metabolites, arachidonic acid achieved the highest AUC of 0.85 (95% CI 0.70–1.00), with a sensitivity of 0.72 and a specificity of 0.78 (Fig. 6b). For urine cytokines, the largest AUC was for IL-6 at 0.82 (95% CI 0.63 ~ 1.02), and at the optimal cutoff point, the sensitivity was 0.77, and specificity was 0.78 (Fig. 6c). When combining either two or three of them, the largest AUC reached 0.96 (95% CI 0.86–1.06), with a sensitivity of 0.94 and a specificity of 1.00 (Fig. 6d). Additional details on the parameters of these ROC curves can be found in Supplementary Table 4. However, there was no significant differences in the AUC among any of the two combinations (Delong test, all *p* > 0.05) (Table 3).

**Table 3:** Comparisons of ROC curves of the combined markers

Curve 1	Curve 2	SE	95% CI	Z value	*p-*value
*Actinomycetaceae* + Arachidonic acid	*Actinomycetaceae* + Arachidonic acid + IL-6	0.03	− 0.05 to 0.10	0.673	0.50
*Actinomycetaceae* + Arachidonic acid	*Actinomycetaceae* + IL-6	0.05	− 0.09 to 0.11	0.123	0.90
*Actinomycetaceae* + Arachidonic acid	Arachidonic acid + IL-6	0.05	− 0.09 to 0.09	0.000	1.00
*Actinomycetaceae* + IL-6	*Actinomycetaceae* + Arachidonic acid + IL-6	0.02	− 0.01 to 0.08	1.299	0.19
*Actinomycetaceae* + IL-6	Arachidonic acid + IL-6	0.03	− 0.06 to 0.07	0.178	0.85
Arachidonic_acid + IL-6	*Actinomycetaceae* + Arachidonic acid + IL-6	0.03	− 0.03 to 0.08	0.833	0.40

SE: standard error; CI: confidence interval; IL: interleukin.* p* was calculated by Delong test

## Discussion

In the present study, we observed an upregulated diversity of the urinary microbiome and inflammatory cytokines in patients with bladder cancer compared to cancer-free participants. Also, the abundances of fatty acids and fatty acylcarnitines increased in bladder cancer patients, with the latter declining after tumor resection. The abundances of certain feature microbiota were positively correlated with the levels of inflammatory cytokines, fatty acids and fatty acylcarnitines. Functional enrichment analysis revealed that the glyoxylate cycle and fatty acid metabolism might be potential pathways involved in the pathogenesis of bladder cancer. In addition, the combination of biomarkers from urinary microbiome, metabolome, and cytokines, either two or three of them, displayed excellent diagnostic efficiency for predicting bladder cancer. Specifically, the panel of *Actinomycetaceae* + Arachidonic acid + IL-6 showed promise as noninvasive screening and diagnostic tools for bladder cancer.

The findings on the urinary microbiome in bladder cancer have been inconsistent across various studies. Some studies [12, 14, 30] reported a significant higher α diversity evenness index in bladder cancer patients compared to controls, while others found no significant differences [13, 31]. Such discrepancies among studies might be attributed to variations in study design, sampling procedures and sample types. In this study, we observed a higher α diversity of urinary microbiome in bladder cancer using mid-stream urine samples, which might be the result of urinary tract infection history of bladder cancer patients. At genus level, we found increased abundances of *Acinetobacter, Actinomyces, Corynebacterium, Micrococcus, Staphylococcus, Streptococcus* and *Tepidomonas* in bladder cancer urine, which is consistent with several other studies [32–34]. The dysbiosis of the local microbial environment has been associated with the development of various epithelial cancers [35, 36], suggesting that altered urinary microbiome might also play a role in the pathogenesis of bladder cancer by inducing a sustained inflammatory microenvironment [37, 38]. It is known that microbe- or pathogen-associated molecular patterns (MAMPs or PAMPs), including bacteria cell wall component lipopolysaccharide (LPS), can be recognized by Toll-like receptors (TLRs) and subsequently activate inflammation-related signaling pathways, leading to the expression of inflammatory cytokines such as IL-6 and IL-8. These inflammatory cytokines have been reported to be pro-tumorigenic in bladder cancer [39, 40]. In addition, genotoxins, including reactive oxygen species (ROS) and hydrogen sulfide (H2S), can be released from host immune cells and microbes and contribute to DNA damage and elicit tumor development [41–44]. In the present study, elevated levels of IL-6, IL-8, and IL-10 were observed in the cancerous group and the IL-6 were positively correlated with the relative abundance of the feature microbiota. This indicated that a proinflammatory environment induced by the dysbiosis of microbiome might potentially result in carcinogenesis in the Before group. Moreover, after tumor resection, there was no obvious variance in the diversity and component of urinary microbiome or the levels of cytokines, indicating a potential risk for recurrence. It is important to note that the urinary microbiome comprises taxa from multiple sites within the urinary system, including the surface of the urethral and ureteral tract, kidney, or inside the tumor [16]. suggesting that tumor-associated microbiome only accounted for a small portion of the whole urinary microbiome. Therefore, the removal of tumor-associated microbiome had little effect on the urinary microbiome and inflammatory cytokines, indicating that urinary microbiome dysbiosis might be a causal factor, rather than a consequence, of the development of bladder cancer.

It is well-documented that microbiota metabolism may also contribute to intensifying the proinflammatory microenvironment in the bladder and exacerbating damage by producing genotoxins like acetaldehyde, thereby influencing host cell metabolism [45, 46]. Emerging evidence showed that alterations in the profile of urinary metabolome and the identification of metabolic biomarkers have shown promise for diagnosing bladder cancer [21, 22, 25, 47]. However, many of these were cross sectional studies and their findings were largely inconsistent. Recently, an animal experiment that integrated unbiased metabolomics, lipidomics, and transcriptomics analyses with bladder cancer tissues, suggested that impaired fatty acid beta-oxidation (FAO) was pivotal to the progression of low grade to high grade bladder cancer [48]. Consistently, we also found FAO metabolic pathway was enriched between pre- and post-operation groups. In addition, by comparing metabolites between the healthy individuals and bladder cancer patients, we identified two significant pathways that might potentially involved in the bladder cancer formation, namely glycine, serine, and threonine metabolism, as well as glyoxylate and dicarboxylate metabolism. Glyoxylate cycle, which serves as a substitute of the tricarboxylic acid (TCA) cycle, occurs in plants and microbes instead of human beings due to the absence of key enzymes, malate synthase (MS) and isocitrate lyase (ICL) [49–51]. Thus, it is plausible that the differentially expressed metabolites between the non-cancerous and bladder cancer group might be a result of dysbiosis of urinary microbiome. What’s more, in certain bacterial and fungi, the glyoxylate cycle enables the conversion of acetate and acetyl-CoA, produced during FAO, into succinate and finally converted into carbohydrates [49, 52]. This explained the relatively lower levels of succinate and significantly increased levels of fatty acids in the bladder cancer group compared to the control group. Therefore, we presume that due to the urinary microbiome dysbiosis, the glyoxylate cycle was downregulated, leading to an impaired conversion of fatty acids into carbohydrates, which ultimately lead to a cumulation of fatty acids and decreased level of carbohydrates in the urine of bladder cancer patients. On the other hand, fatty acids are important sources for energy production and membrane biogenesis in tumor cells, and abnormal FAO is involved in various aspects of carcinogenesis [53, 54]. Before oxidation, fatty acids need to be shuttled into mitochondria (very long chain fatty acids shortened in peroxisome first) with collaboration of a series of enzymes, including carnitine palmitoyltransferase I (CPT1) and carnitine palmitoyltransferase II (CPT2) that converts and reconverts fatty acyl-CoA and fatty acylcarnitines (Fig. 5a). In the present study, the concentrations of carnitine and fatty acylcarnitines in the Before group were remarkably elevated but declined after tumor removal, suggesting an abnormality of FAO in bladder cancer cells. These suggested a potential connection between abnormal microbial metabolism and tumor metabolism, possibly mediated by fatty acids metabolism.

Finally, the biomarkers identified in this study were utilized to perform ROC curves to assess their potential as noninvasive diagnostic tools for bladder cancer. In fact, the diagnostic performance of metabolites in detecting bladder cancer has been studied extensively, with the sensitivity ranging from 27 to 100%, while specificity between 43 and 96% [55]. Our results showed that the biomarker panels exhibited superior diagnostic performance, achieving sensitivity levels between 86 to 94% and specificity ranging from 89 to 100% in distinguishing bladder cancer patients from non-cancerous individuals. However, further validation in a large cohort is necessary to confirm these findings.

Limitations are inevitable in this study. First, the sample size is relatively small with an obvious difference between the bladder cancer and the control groups. Second, a validation cohort is necessary to confirm the distinguishing performance of the biomarker panel. Finally, this study only presented observations and assumptions of the underlying network involved in this phenomenon. Further comprehensive experiments are needed to substantiate these findings.

## Conclusions

These findings demonstrated a close relationship between urinary microbiome, inflammation environment, and perturbed fatty acid metabolism in bladder cancer, which can throw light on the exploration of potential pathogenesis and development of noninvasive diagnostic tools as well as novel targets for bladder cancer.

### Supplementary Information

Supplementary Material 1.

## Data Availability

The raw reads of 16S rRNA sequencing are available NCBI (https://www.ncbi.nlm.nih.gov/) at accession number PRJNA1104398. Other datasets generated during the current study are available from the corresponding authors upon reasonable request.
