# Supplementary material for: Urinary microbiome dysbiosis is associated with an inflammatory environment and perturbed fatty acids metabolism in the pathogenesis of bladder cancer
Source: J Transl Med. 2024 Jul 5;22:628. doi: 10.1186/s12967-024-05446-7 (PMC11227203; doi:10.1186/s12967-024-05446-7)
Supplement: Supplementary file 1 — Supplementary Material 1. [file 12967_2024_5446_MOESM1_ESM.docx]

**Contents**

Supplementary figure 1 Page 2

Supplementary figure 2 Page 2

Supplementary figure 3 Page 3

Supplementary table 1 Page 4

Supplementary table 2 Page 4

Supplementary table 3 Page 4

Supplementary table 4 Page 5


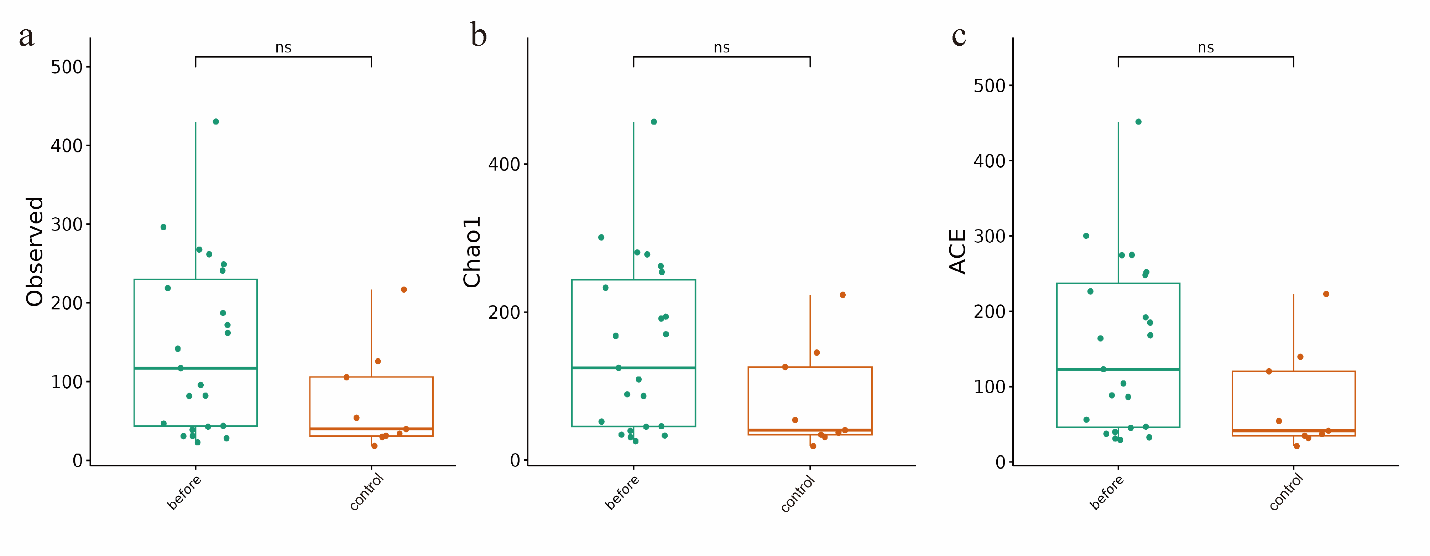


**Supplementary figure 1. Other α indexes of urinary microbiome.** There were no significant differences in Observed species (a), Chao1 (b), and ACE (c) indexes between the Before group and the Control group.


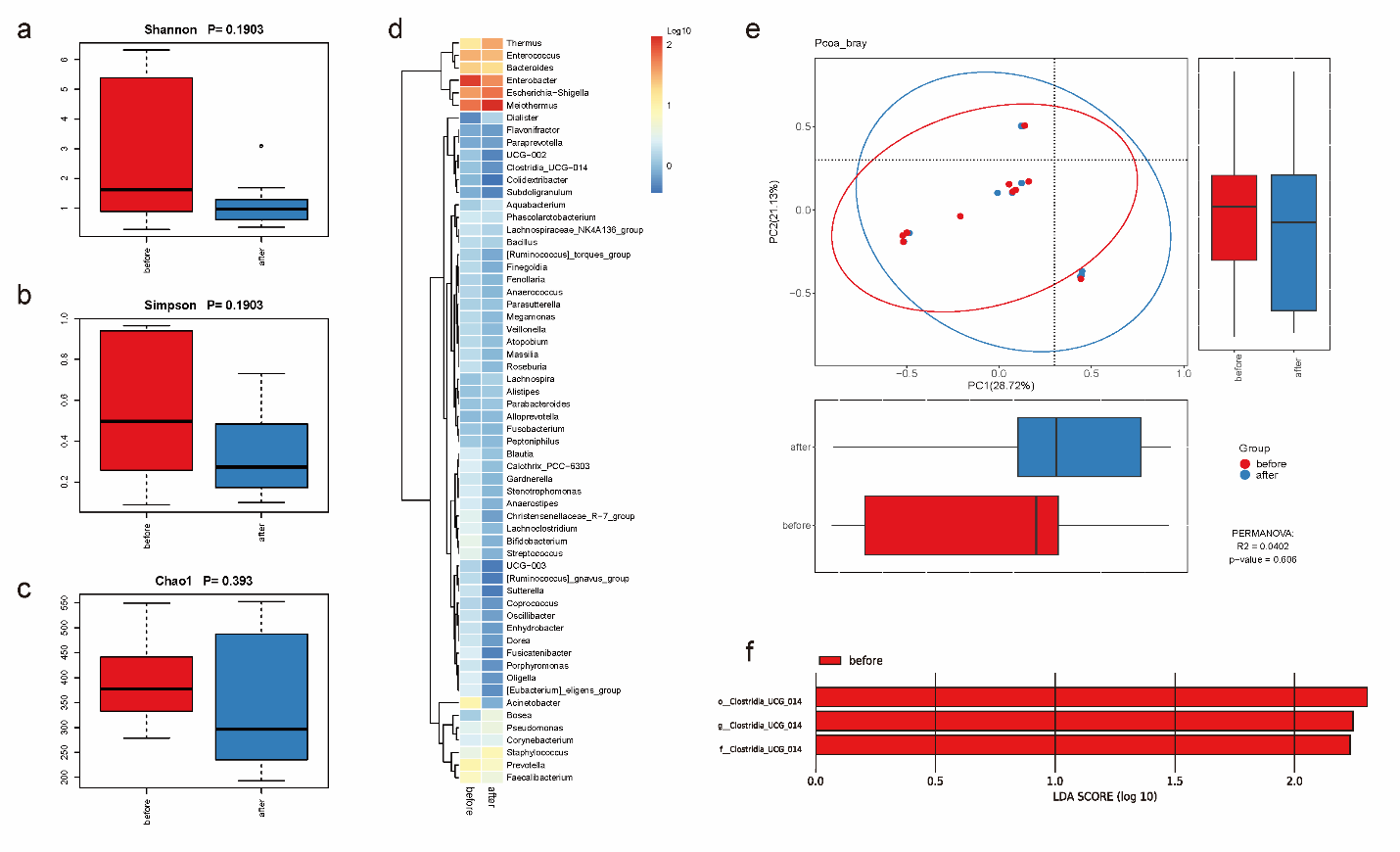


**Supplementary figure 2. Urine microbiome changes after tumor resection.** (a) Shannon index, Simpson index (b), and Chao1 index (c) indicated no significant differences in α diversity of urine microbiome between the Before group and the After group (Wilcoxon test *p*>0.05; *p*>0.05; *p*>0.05); (d) Comparison of the relative abundance of microbiota between two groups; (e) PCOA model showed there was no significant difference in β diversity between two groups; (f) LEfSe analysis identified *Clostridia_UCG_014* to be feature microbiota for the Before group.


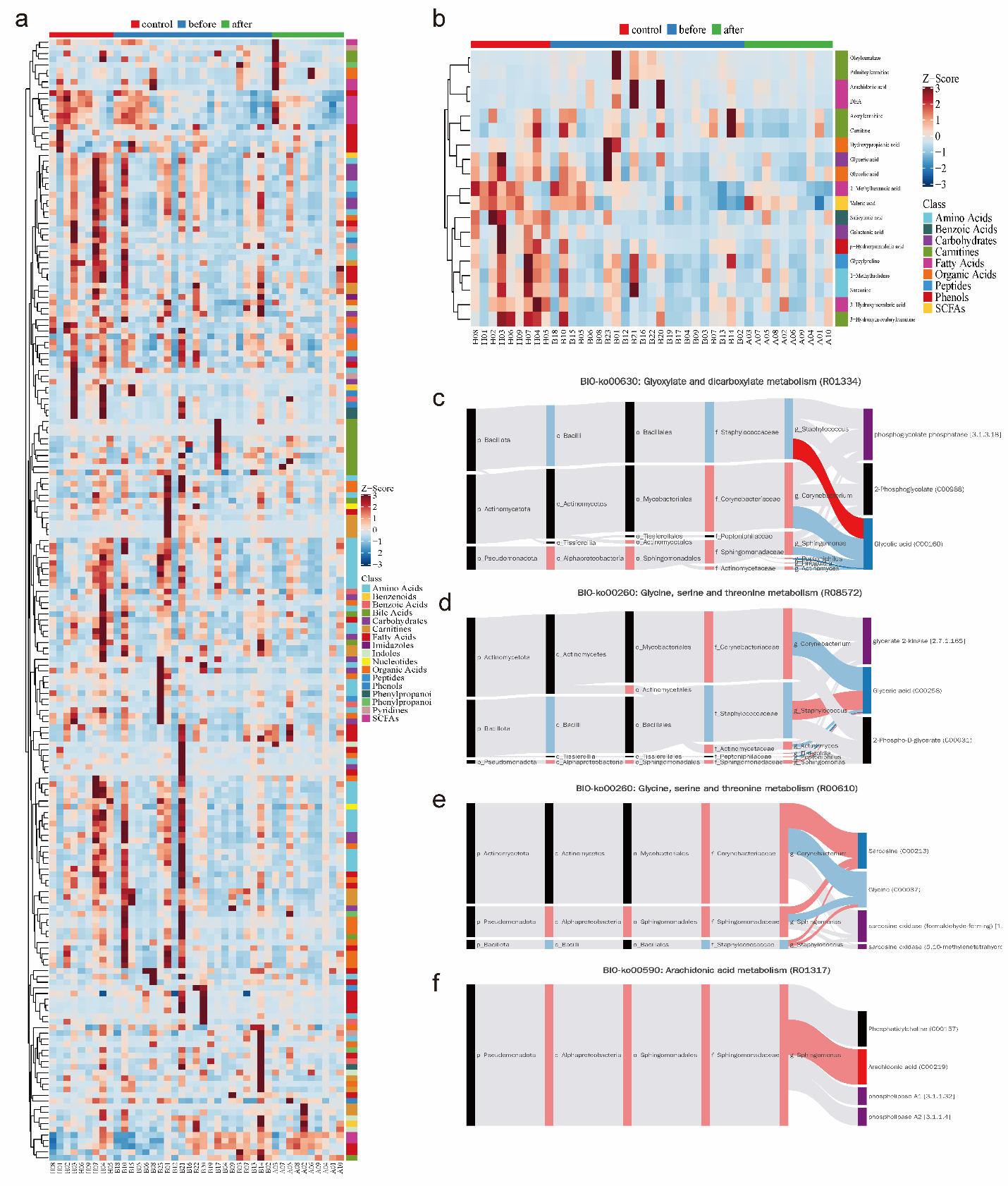


**Supplementary figure 3. Metabolites and correlations with microbiota.** Heatmaps of all metabolites (a) and the differentially expressed metabolites (b) of all samples. The Sankey network for microbiota-metabolic reaction in Glyoxylate and dicarboxylate metabolism (c), Glycine, serine, and threonine metabolism (d, e), and Arachidonic acid metabolism (f).

**Supplementary table 1. UPLC-MS/MS instrument settings.**

| **UPLC** |  |
| --- | --- |
| Column | ACQUITY UPLC BEH C18 1.7 µM VanGuard pre-column (2.1×5 mm) and ACQUITY UPLC BEH C18 1.7 µM analytical column (2.1 × 100 mm) |
| Column Temp. (°C) | 40 |
| Sample Manager Temp. (°C) | 10 |
| Mobile Phases | A=water with 0.1% formic acid; and B=acetonitrile / IPA (70:30) |
| Gradient Conditions | 0-1 min (5% B), 1-11min (5-78% B), 11-13.5 min (78-95% B), 13.5-14 min (95-100% B), 14-16 min (100% B), 16-16.1 min (100-5% B), 16.1-18 min (5% B). |
| Flow Rate (mL/min) | 0.40 |
| Injection Vol. (µl) | 5.0 |
| MASS SPECTROMETER | |
| Capillary (Kv) | 1.5 (ESI+), 2.0 (ESI-) |
| Source Temp (°C) | 150 |
| Desolvation Temp (°C) | 550 |

UPLC: ultra-performance liquid chromatography; MS: mass spectrometry.

**Supplementary table 2. Urine cytokines change before and after treatment.**

|  | **Before group (n=10)** | **After group (n=10)** | ***p-value*** |
| --- | --- | --- | --- |
| IL-1β | 21.76±19.74 | 20.88±21.58 | 0.94 |
| IL-2 | 1.93±1.00 | 1.20±0.95 | 0.20 |
| IL-4 | 9.31±5.66 | 6.12±3.42 | 0.22 |
| IL-5 | 10.94±4.45 | 7.21±2.56 | 0.08 |
| IL-6 | 13.81 (7.34, 35.64) | 28.66 (5.79, 173.33) | 0.58* |
| IL-8 | 169.28±358.49 | 199.16±325.63 | 0.85 |
| IL-10 | 7.01±4.26 | 4.09±4.24 | 0.15 |
| IL-12p70 | 17.06±13.93 | 10.94±9.78 | 0.37 |
| IL-17 | 25.52±17.23 | 19.94±12.68 | 0.52 |
| TNF-α | 6.82±5.09 | 2.95±2.66 | 0.07 |
| IFN-α | 1.85±1.67 | 1.29±1.23 | 0.43 |
| IFN-γ | 5.96±3.36 | 3.76±4.75 | 0.25 |

IL: interleukin; TNF: tumor necrosis factor; IFN: interferon. **p* was calculated by the paired sample Wilcoxon signed-rank test, others were calculated by paired t-test.

**Supplementary table 3. Urine cytokines change between the Control group and the After group.**

|  | **Control group (n=9)** | **After group (n=10)** | ***p-value*** |
| --- | --- | --- | --- |
| IL-1β | 8.30 (4.74, 48.04) | 14.53 (4.05, 30.35) | 1.00 |
| IL-2 | 1.42±0.75 | 1.20±0.95 | 0.59 |
| IL-4 | 7.44±5.67 | 6.12±3.42 | 0.54 |
| IL-5 | 7.97±3.69 | 7.21±2.56 | 0.60 |
| IL-6 | 6.36 (5.39, 8.26) | 28.66 (6.97, 173.33) | 0.01 |
| IL-8 | 6.38 (3.44, 23.89) | 75.05 (3.33, 273.19) | 0.19 |
| IL-10 | 3.65±1.92 | 2.32 (1.06, 7.12) | 0.68 |
| IL-12p70 | 6.99 (3.42, 28.32) | 10.94±9.78 | 0.51 |
| IL-17 | 20.18±16.22 | 19.94±12.68 | 0.97 |
| TNF-α | 4.32±3.70 | 2.95±2.66 | 0.36 |
| IFN-α | 1.02±0.51 | 1.29±1.23 | 0.56 |
| IFN-γ | 3.81±2.62 | 3.76±4.75 | 0.41 |

IL: interleukin; TNF: tumor necrosis factor; IFN: interferon. *p* was calculated by Student’s t-test or Mann-Whitney U test.

**Supplementary table 4. Area under the curve of ROC curves.**

|  | **AUC** | **SE** | **95% LCI** | **95% UCI** |
| --- | --- | --- | --- | --- |
| p_Planctomycetota | 0.69 | 0.11 | 0.46 | 0.92 |
| c_Phycisphaerae | 0.67 | 0.11 | 0.44 | 0.90 |
| o_Actinomycetales | 0.82 | 0.09 | 0.64 | 1.00 |
| o_Staphylococcales | 0.75 | 0.11 | 0.52 | 0.98 |
| o_Peptostreptococcales_Tissierellales | 0.73 | 0.11 | 0.50 | 0.97 |
| o_Tepidisphaerales | 0.67 | 0.11 | 0.44 | 0.90 |
| f_Actinomycetaceae | 0.82 | 0.09 | 0.64 | 1.00 |
| f_Corynebacteriaceae | 0.73 | 0.12 | 0.49 | 0.98 |
| f_Staphylococcaceae | 0.75 | 0.11 | 0.52 | 0.99 |
| f_Peptostreptococcales_Tissierellales | 0.77 | 0.11 | 0.54 | 1.00 |
| f_Tepidisphaeraceae | 0.67 | 0.11 | 0.44 | 0.90 |
| g_Actinomyces | 0.75 | 0.11 | 0.53 | 0.97 |
| g_Corynebacterium | 0.73 | 0.12 | 0.49 | 0.98 |
| g_Micrococcus | 0.67 | 0.11 | 0.44 | 0.90 |
| g_Staphylococcus | 0.75 | 0.11 | 0.52 | 0.99 |
| g_Finegoldia | 0.75 | 0.11 | 0.53 | 0.97 |
| g_Peptoniphilus | 0.78 | 0.10 | 0.58 | 0.98 |
| g_Negativicoccus | 0.69 | 0.11 | 0.46 | 0.92 |
| g_Tepidisphaera | 0.67 | 0.11 | 0.44 | 0.90 |
| g_Asticcacaulis | 0.72 | 0.11 | 0.50 | 0.94 |
| g_Methylobacterium_Methylorubrum | 0.76 | 0.11 | 0.54 | 0.97 |
| IL-6 | 0.82 | 0.09 | 0.63 | 1.02 |
| IL-8 | 0.74 | 0.11 | 0.52 | 0.95 |
| IL-10 | 0.76 | 0.10 | 0.56 | 0.96 |
| Arachidonic acid | 0.85 | 0.07 | 0.70 | 1.00 |
| DHA | 0.79 | 0.10 | 0.59 | 0.99 |
| Palmitoylcarnitine | 0.74 | 0.10 | 0.54 | 0.95 |
| Oleylcarnitine | 0.77 | 0.10 | 0.56 | 0.97 |
| Actinomycetaceae+IL-6 | 0.93 | 0.06 | 0.80 | 1.06 |
| Actinomycetaceae+Arachidonic acid | 0.93 | 0.05 | 0.83 | 1.04 |
| Arachidonic acid+IL-6 | 0.91 | 0.07 | 0.77 | 1.06 |
| Actinomycetaceae+Arachidonic acid+IL-6 | 0.96 | 0.05 | 0.86 | 1.06 |

ROC: Receiver operating characteristic curve; IL: interleukin; TNF: tumor necrosis factor; IFN: interferon; DHA: Docosahexaenoic Acid; AUC: area under the curve; SE: standard error; LCI: lower confidence interval; UCI: upper confidence interval.
